# Integrating Genomics with Nutrition Models to Improve the Prediction of Cattle Performance and Carcass Composition under Feedlot Conditions

**DOI:** 10.1371/journal.pone.0143483

**Published:** 2015-11-24

**Authors:** Luis O. Tedeschi

**Affiliations:** Department of Animal Science, Texas A&M University, College Station, Texas, United States of America; Wageningen UR Livestock Research, NETHERLANDS

## Abstract

Cattle body composition is difficult to model because several factors affect the composition of the average daily gain (**ADG**) of growing animals. The objective of this study was to identify commercial single nucleotide polymorphism (**SNP**) panels that could improve the predictability of days on feed (**DOF**) to reach a target United States Department of Agriculture (**USDA**) grade given animal, diet, and environmental information under feedyard conditions. The data for this study was comprised of crossbred heifers (n = 681) and steers (n = 836) from commercial feedyards. Eleven molecular breeding value (**MBV**) scores derived from SNP panels of candidate gene polymorphisms and two-leptin gene SNP (UASMS2 and E2FB) were evaluated. The empty body fat (**EBF**) and the shrunk body weight (**SBW**) at 28% EBF (**AFSBW**) were computed by the Cattle Value Discovery System (**CVDS**) model using hip height (EBF_HH_ and AFSBW_HH_) or carcass traits (EBF_CT_ and AFSBW_CT_) of the animals. The DOF_HH_ was calculated when AFSBW_HH_ and ADG_HH_ were used and DOF_CT_ was calculated when AFSBW_CT_ and ADG_CT_ were used. The CVDS estimates dry matter required (**DMR**) by individuals fed in groups when observed ADG and AFSBW are provided. The AFSBW_CT_ was assumed more accurate than the AFSBW_HH_ because it was computed using carcass traits. The difference between AFSBW_CT_ and AFSBW_HH_, DOF_CT_ and DOF_HH_, and DMR and dry matter intake (**DMI**) were regressed on the MBV scores and leptin gene SNP to explain the variation. Our results indicate quite a large range of correlations among MBV scores and model input and output variables, but MBV ribeye area was the most strongly correlated with the differences in DOF, AFSBW, and DMI by explaining 8, 13.2 and 6.5%, respectively, of the variation. This suggests that specific MBV scores might explain additional variation of input and output variables used by nutritional models in predicting individual animal performance.

## Introduction

A recent survey of U.S. consumers regarding meat quality indicated a greater satisfaction and preference for United States Department of Agriculture (**USDA**) Choice or Canadian AAA graded meat versus lower quality grade meat (i.e., USDA Selected and Canada AA) [1], but typically about only 60% of the cattle in the United States grade USDA Choice or higher [2]. Nutrition and growth models can assist in the management of feedlot cattle by grouping animals that are likely to reach a common carcass composition when fed a diet for a given period. This management strategy has the potential to decrease the heterogeneity of the carcasses among animals within a pen and to assist in the decision making process of when to slaughter animals, which typically leads to increased profitability. Several growth models are available [3–9], but only two of them have been developed for this specific purpose [8, 9]. An evaluation of four growth models indicated that they differ in the predictions of average daily gain (**ADG**), dry matter intake (**DMI**), and body and carcass composition likely because the assumptions and definitions used during their development were different among them [10]. The Cattle Value Discovery System (**CVDS)** [8] is an applied model that predicts the performance and carcass composition of individual animals based on information regarding the animal, diet, and the environment as recommended by the National Research Council (**NRC)** [11] and the Cornell Net Carbohydrate and Protein System [12]. Phenotypic and genetic evaluations of the CVDS have resulted in high correlations (r > 0.80) between observed and predicted DMI [13, 14] and feed-to-gain ratio (i.e., feed efficiency) [15, 16]. Despite these high correlations under controlled conditions, several assumptions and inputs can affect the prediction outcome, including mature size [17].

It is likely the determination of the body weight (**BW**) at a given body composition is one of the most influential variables in accurately predicting animal requirements for growth and body/carcass composition. Body composition is, however, mathematically difficult to predict by nutrition models because several factors can affect the composition of the weight gain of growing beef cattle. Hence, the information of genetic predisposition for growth and development could be useful in improving the accuracy in determining the shrunk BW (**SBW**) at 28% empty body fat (**AFSBW**) for individuals. The use of commercial single nucleotide polymorphism (**SNP**) panels to predict animal performance and carcass traits have yielded promising, but yet yields variable, results [18–20]. It is possible that coefficients of complex model parameters may contain unexplained genetic variation [21]; thus, unbiased coefficients could be obtained if genetic variation is explicitly accounted for. Perhaps the combination of SNP with nutrition models could improve the predictions of animal performance and provide more reliable tools to assess the profitability of cattle fed under feedlot conditions by meeting consumer preferences. The objective of this study was to identify molecular breeding values (**MBV**) obtained from commercial SNP panels that can be used to improve the CVDS prediction of days on feed (**DOF**) to reach a target USDA grade and allocation of feed among individuals in a pen given animal, diet, and environmental information under feedyard conditions.

## Materials and Methods

A specific Animal Use Protocol was not obtained for this study because no animals were used. We analyzed data provided by Decatur County Feedyard (Decatur, KS) and Merial/IGENITY (Duluth, GA). A detailed description of data collection and processing was provided previously [19]. The procedures for the Care and Use of Agricultural Animals in Research and Teaching outlined by the Federation of Animal Science Societies [22] for live animals were observed by them.

### Animal Database

The data for this study was collected in the spring of 2004. Table 1 lists the descriptive statistics of the database for the crossbred heifers (n = 681) and steers (n = 836). Animals were managed according to the commercial feedyard’s practices. Steers received a combination implant (initial BW ≤ 340 kg: Revalor S; initial BW >340 kg: Revalor IS). Heifers with initial BW < 286 kg received Revalor 200 (Merck Animal Health, Whitehouse Station, NJ) while heifers with initial BW > 286 kg were implanted with 200 mg of trenbolone acetate (Finaplix-H, Merck Animal Health, Whitehouse Station, NJ). Animals were slaughtered in a commercial abattoir and carcass traits were obtained, including hot carcass weight (**HCW**), USDA quality grade (**QG**) and yield grade (**YG**), and ribeye area (**REA**). The REA was measured by an electronic image capture and analysis system (VBG2000 Grading System, Vision-For-You Inc., Dakota Dunes, SD). The fat thickness (**FT**; i.e., backfat) was determined using an ultrasound machine the week before slaughter.

**Table 1:** Descriptive statistics of the database for heifers and steers (N = 1,517).

Variables^1^	Heifers				Steers			
	n	Mean	SD	Range	n	Mean	SD	Range
Performance								
Initial BW, kg	681	297	39.7	182 to 427	836	314	45.6	166 to 440
Hip height, cm	681	117	5.01	104 to 144	836	120	4.99	104 to 147
Final BW, kg	681	507	54.9	364 to 654	836	556	50.6	386 to 704
DOF, d	681	161	34.9	91 to 262	836	162	42.7	91 to 285
ADG, kg/d	681	1.36	0.312	0.514 to 2.23	836	1.57	0.414	0.662 to 3.04
Carcass traits								
HCW, kg	681	326	33.8	206 to 430	836	356	34.1	248 to 441
Backfat, cm	681	1.25	0.46	0 to 2.90	836	1.14	0.40	0 to 2.41
REA, cm^2^	681	85.4	10.95	53.6 to 137	836	88.3	10.4	58.1 to 123
Marbling^2^	681	4.85	1.12	3 to 9	836	4.51	1	3 to 9
USDA YG	654	2.57	0.68	1 to 4	798	2.51	0.63	1 to 4
IGENITY MBV								
CAB marbling	681	49.1	16.6	1.94 to 97.7	836	52.3	17.9	3.17 to 101.2
ADG	681	0.15	0.08	-0.11 to 0.37	836	0.15	0.08	-0.09 to 0.45
HCW	681	27.2	8.49	-16.1 to 48.6	836	26.3	9.01	-17.7 to 53.1
REA	681	-0.36	0.47	-2.01 to 0.83	836	-0.42	0.47	-1.55 to 0.87
50 SNP marbling	681	-30.4	21.9	-82.9 to 38.4	836	-25.6	22.7	-96.9 to 38.1
50 SNP REA	681	-0.56	0.67	-2.37 to 1.5	836	-0.62	0.65	-2.16 to 1.64
All Marbling	632	80.7	20.4	25.2 to 148	800	84.1	22.2	19.8 to 145
All REA	635	1.11	0.31	0.13 to 2.15	796	1.11	0.31	0.27 to 2.09
All ADG	634	0.38	0.10	0.04 to 0.64	799	0.40	0.10	0.04 to 0.69
All RFI	636	2.18	1.93	-4.29 to 8.53	804	2.25	1.80	-3.62 to 9.61
*Bos taurus* RFI	636	1.98	0.60	0.07 to 3.74	803	2.05	0.57	0.45 to 3.73
Leptin SNP	Frequency (CC:CT:TT)			*P*-value^3^	Frequency (CC:CT:TT)			*P*-value^3^
E2FB	254:416:164			< 0.0001	233:333:113			< 0.0001
UASMS2	406:349:61			< 0.0001	313:282:70			< 0.0001

^1^ DOF = days on feed; REA = ribeye area; USDA YG = USDA Yield Grade; MBV = molecular breeding value; SNP = single nucleotide polymorphism.

^2^ Marbling scores for USDA quality grades Standard (2 = practically devoid or 3 = traces), Select (4 = slight), low Choice (5 = small), Choice (6 = modest), high Choice (7 = Moderate), low Prime (8 = slightly abundant), and Prime (9 = moderately abundant).

^3^ *P*-value of χ^2^ test for frequency percent of 25:50:25 for CC, CT, and TT leptin genotypes, respectively.

### Molecular Breeding Value Scores

The MBV scores used in the evaluation were derived from SNP panels of candidate gene polymorphisms. Routine screening programs were conducted in SNP discovery populations to identify SNP in physiological and candidate genes and QTL whose effects were expected to influence growth and composition in feedlot cattle [23–25]. Identified SNP were then incorporated into genotyping panels run on the Sequenom MassArray (Sequenom, Inc, 3595 John Hopkins Court, San Diego, CA, 92121; http://www.sequenom.com; accessed on September 6, 2015), using DNA from animals with appropriate phenotypes, including raw phenotypes or estimated breeding value (**EBV**). From the database of all SNP markers genotyped in the discovery population, a univariate analysis was conducted to evaluate the association between the individual markers and the trait, based on a regression of the number of copies of one of the alleles on the trait. Those SNP that showed significant allele substitution effects for each of several key traits were selected and included into a stepwise regression model that evaluates markers sequentially in an additive model for each trait [26]. The SNP that were significant when considered simultaneously were included into the final model and the additive genetic effect for each marker was summed to create the MBV scores [27]. Once the significant SNP were identified and the allele substitution effects determined in a stepwise model, an independent validation population of animals was genotyped and MBV scores generated to verify the predictive ability of the SNP panel for each trait. The following MBV scores were investigated: **MBV**_**CABMRB**_ is a panel of approx. 90 SNP that is trained on the trait of marbling of Angus cattle (an elevated MBV_CABMRB_ is associated with increased marbling and backfat and reduced REA); **MBV**_**ADG**_ is an MBV that was trained on the trait of ADG (an increased MBV_ADG_ value should be associated with increased ADG); **MBV**_**HCW**_ is an MBV that was trained on the trait of HCW (an increased MBV_HCW_ value should be associated with increased HCW); **MBV**_**REA**_ is an alternate MBV for REA (an increased MBV_REA_ values should be associated with increased REA); **MBV**_**50SNPMRB**_ is an alternate SNP panel trained on the trait of marbling; unlike the MBV_CABMRB_, the MBV_50SNPMRB_ uses only 50 SNP (an increased MBV_50SNPMRB_ values should be associated with increased marbling); **MBV**_**50SNPREA**_ is an alternate SNP panel trained on the trait of REA that uses only 50 SNP (an increased MBV_50SNPREA_ values should be associated with increased REA); **MBV**_**AllMRB**_ is an alternative marbling MBV that was built from a crossbred sire repository; **MBV**_**AllREA**_ is an alternative REA MBV that was built from a crossbred sire repository; **MBV**_**AllADG**_ is another ADG MBV that was built from a crossbred sire repository; **MBV**_**AllRFI**_ is an MBV for residual feed intake (**RFI**) designed for use across breeds, including *Bos indicus*-influenced cattle; and **MBV**_**BTRFI**_ is an MBV for RFI for *Bos taurus* cattle (Table 1).

The specific leptin gene SNP analysis for this database was described previously [19]. Briefly, a hair was plucked from each animal for determination of two leptin SNP: UASMS2 [28] and E2FB [29]. The hair samples were genotyped by IGENITY (Merial Ltd., Atlanta, GA) at a commercial genotyping facility. The allelic frequencies of these SNP are shown in Table 1. The nine possible genotype combinations of UASMS2 and E2FB SNP (CCCC, CCCT, CCTT, CTCC, CTCT, CTTT, TTCC, TTCT and TTTT) were used to create a leptin SNP classificatory variable.

### Calculations

All simulations were done with CVDS version 1.0.32 using the exponential decay adjustment for composition of the gain [8], and the monthly averages for temperature, relative humidity, and wind speed for Oberlin, KS were used in predicting maintenance requirements of the animals. Specific ingredient information for the diet fed to the animals in this data base was not available; therefore, it was assumed a constant value for diet metabolizable energy (**ME**) of 3.2 Mcal/kg; which is consistent with reported ME values for this feedlot [30]. This diet ME concentration is also consistent with the average net energy for growth (**NEg**) of 1.5 Mcal/kg reported by 29 nutritionists in a recent survey [31], who represent approx. 69% of the cattle on feed in the United States. Previous CVDS simulations suggested a linear relationship, without any interaction, between the adequacy of the CVDS in predicting animal DMI and different concentration values for the diet ME [32]. Therefore, even if the diet ME of 3.2 Mcal/kg was not correct, an over- or underprediction of the diet ME would modify the CVDS adequacy, but the change would be relatively constant among different dietary ME values.

The empty body fat (**EBF**) and the AFSBW can be computed by the CVDS using either the hip height (**EBF**_**HH**_) or carcass traits (**EBF**_**CT**_) of the animals. For projection purposes, hip height (**HH**), BW, and body condition score (**BCS**) are generally the variables available when the animals are sorted upon arriving at feedyards. Carcass traits [30] or ultrasound information [33] could provide a better estimate of EBF, but they are not always available. Thus, AFSBW predicted using carcass traits was compared with that predicted using HH. The AFSBW predicted with carcass traits (**AFSBW**_**CT**_) was assumed the dependent variable (Y) while the AFSBW predicted with the HH (**AFSBW**_**HH**_) was the independent variable (X). The difference between them (**ΔAFSBW** = AFSBW_CT_−AFSBW_HH_) was used to identify independent explanatory variables (i.e., MBV scores and leptin SNP), assuming that AFSBW_CT_ would more accurately represent the BW at 28% EBF.

#### Predicting AFSBW from hip height

Hip height was used to compute frame score by using the equations published by the Beef Improvement Federation [34] for animals between the ages of 5 and 21 mo, as shown in Eq (1) for bulls and Eq (2) for heifers. The AFSBW_HH_ was then computed from frame score using Eq (3). The data used to derive Eq (3) was based on three frame size classifications of feeder cattle when they reached the USDA low Choice grade, assumed to be at 28% EBF, that was originally available [35] and converted to equivalent SBW [36]. Eq (3) is the result of the linear regression of equivalent SBW on frame score [36].

$$\begin{array}{l} FS_{Bull}=-11.548+0.1920\times HH-0.0289\times Age+0.00001947\times Age^{2}+\\ 0.00001315\times HH\times Age \end{array}$$ (1)

$$\begin{array}{l} FS_{Female}=-11.7086+0.1859\times HH-0.0239\times Age+0.0000146\times Age^{2}+\\ 0.00002988\times HH\times Age \end{array}$$ (2)

$$AFSBW_{HH}=\{\begin{array}{c} 40\times FS_{Bull}+440\\ 33.4\times FS_{Steer}+366.6\\ 26.7\times FS_{Female}+293.2 \end{array}$$ (3)

Where FS is frame score, 1 to 9 scale; HH is hip height, cm; Age is age when HH was determined, d; and AFSBW is adjusted final shrunk BW at 28% empty body fat. Age was assumed 12 mo.

#### Predicting EBF and AFSBW from carcass traits

The EBF was computed either using Eq (4) when REA was available [30] or Eq (5) when REA was not available [8]. The empty BW (EBW) associated with the calculated EBF was computed with Eq (6) [37], which had the best fit in predicting EBW from HCW [8]. Then, AFSBW_CT_ was computed using the 14.26 kg/% EBF relationship [30] as shown in Eq (7). The 0.891 is the conversion factor between EBW and SBW [11].

$$\begin{array}{l} EBF=\;17.76207+4.68142\times FT+0.01945\times HCW+\\ 0.81855\times MS-0.06754\times REA \end{array}$$ (4)

EBF= 14.08796+4.7135×FT+0.01316×HCW+0.90855×MS (5)

EBW=1.316×HCW+32.39 (6)

$$AFSBW_{CT}=\frac{EBW+14.26\times \left(28-EBF\right)}{0.891}$$ (7)

Where EBF is empty body fat, %; FT is fat thickness, cm; HCW is hot carcass weight, kg; MS is marbling score (2 = practically devoid, 3 = traces, 4 = slight, 5 = small, 6 = modest, 7 = Moderate, 8 = slightly abundant, and 9 = moderately abundant); REA is ribeye area, cm^2^; EBW is empty body weight, kg; and AFSBW is adjusted final shrunk body weight at 28% empty body fat, kg.

#### Predicting ADG

The animal characteristics, carcass traits, and diet and environment information were inputted into the CVDS model to predict DMI and ADG as described previously [8]. The ADG predicted when using the AFSBW_HH_ (ADG_HH_) and AFSBW_CT_ (ADG_CT_) were used to compute ΔADG_HH_ (observed ADG minus ADG_HH_) and ΔADG_CT_ (observed ADG minus ADG_CT_). The ΔADG_HH_ and ΔADG_CT_ were regressed on other independent variables (i.e., MBV scores and leptin SNP) in order to identify possible explanatory variables.

#### Predicting expected DOF

Similarly, the DOF_HH_ was calculated when AFSBW_HH_ and ADG_HH_ were used and DOF_CT_ was calculated when AFSBW_CT_ and ADG_CT_ were used as described previously [8]. However, because the accuracy of the DOF_HH_ or DOF_CT_ depends on the accurate prediction of several variables, including DMI, ADG, and AFSBW, an expected DOF (eDOF; Eq (8)) was computed using the observed ADG and assuming that AFSBW_CT_ was the unbiased estimate of the BW at 28% EBF of the animals. Hence, eDOF eliminated the uncertainties in DMI and ADG, but assumed that EBF could be estimated from carcass traits more accurately than HH. Then, ΔDOF_CT_ and ΔDOF_HH_ were calculated as the difference between eDOF and the respective DOF, and they were regressed on possible explanatory variables (i.e., MBV scores and leptin SNP).

$$eDOF=\frac{\left(AFSBW_{CT}-iSBW\right)}{oADG}$$ (8)

Where eDOF is expected days on feed, d; AFSBW_CT_ is the BW at 28% empty body fat, kg; iSBW is initial shrunk BW, kg; and oADG is the observed ADG (shrunk weight basis), kg/d.

#### Predicting dry matter required

Given the animal characteristics, diet, and environment information the CVDS can estimate DM required (DMR) by individuals fed in groups based on observed ADG and AFSBW using a backward calculation technique [8]. A high correlation (r = 0.86) between observed DMI and DMR has been reported [30]. When the CVDS was used to allocate the feed fed to each pen in a commercial feedlot data base containing 12,105 steers and heifers [30], the total observed dry matter consumed was predicted with a bias of less than 1%. Therefore, because individual DMI was not available for this dataset, we assumed the calculated DMR would be a good approximation for the observed DMI. The DMR was computed using the dynamic growth model of the CVDS and AFSBW_CT_. A preliminary analysis indicated a high correlation (r = 0.988) between estimated DMI using either AFSBW_CT_ or AFSBW_HH_, thus the ΔDMI was computed as the difference between DMR and DMI predicted using the AFSBW_HH_. The ΔDMI was then regressed on explanatory variables (i.e., MBV scores and leptin SNP). For comparative purposes, an alternative empirical equation [38] was used to predict DMI. This alternative equation uses animal’s end BW and net energy of the diet available for maintenance (assumed 2.2 Mcal/kg).

### Statistical Analyses

All statistical analyses were conducted with SAS (SAS Inst., Cary, NC). The PROC MEANS and PROC FREQ were used to obtain the descriptive statistics and the χ^2^ test. The PROC CORR was used to obtain pairwise Pearson correlation statistics. The PROC REG was used to perform the regression analysis with the STEPWISE selection option to select the most important independent variables to explain the variation of the dependent variables; interactions and quadratic forms of the independent variables were also investigated. The PROC MIXED was used to evaluate classificatory variables (sex and leptin SNP) using the REML convergence method.

### Adequacy and Cross-Validation Analyses

#### Adequacy evaluation

The adequacy of the equations was evaluated independently to assess precision and accuracy of the predictions using several statistical inferences and measures [39], including the mean square error of prediction (MSEP), the root of MSEP (RMSEP), the MSEP decompositions into mean bias, systematic bias, and random errors [40], the concordance correlation coefficient (CCC) and accuracy (Cb) statistics [41], and the equation precision was assessed via the coefficient of determination (r^2^). Further considerations on model adequacy evaluation have been discussed [42]. Statistical analyses for adequacy were performed with R 3.2 ([43]; http://www.r-project.org; accessed on September 6, 2015) and the Model Evaluation System ([39]; http://nutritionmodels.com/mes.html; accessed on September 6, 2015).

#### Cross validation

The cross-validation technique [44] was used to assess the adequacy of equations with 1,000 random simulations. For each simulation, the database was randomly split into two subsets (k = 2): one subset was the training database to develop the equations (n = 750) and the other subset was the testing database to evaluate the predictions (n = 749). Equations were only fitted to the first subset (k = 1) and the adequacy statistics were calculated using the other subset (k = 2). These equations had the same variables of the selected equations. The cross-validation analysis was based on the cv.glm function of the “boot” package [45] using the general linear model (i.e., ordinary least-squares regression) of R version 3.2 [43]. Adequacy statistics described above were averaged based on the sample size of the k^th^ subset. Only one weighted average of the adequacy statistics was reported for each simulation. Finally, the 2.5% and 97.5% quartiles for CCC, Cb, r^2^, and the decomposition of MSEP were reported.

## Results and Discussion

The SNP frequency of CC, CT, and TT for leptin genotypes was different from the expected frequency of 25, 50, and 25%, respectively (*P* < 0.0001; Table 1) compared to reported frequency [46]. The C allele was predominant over the T allele, but the ratio of C:T was different between the E2FB and UASMS2 SNP (Table 1). For steers, the C allele was 1.24 and 2.46 times more common than the T allele for E2FB and UASMS2 SNP, respectively. Similarly, for heifers the C allele was 1.43 and 2.15 times more common than the T allele for E2FB and UASMS2 SNP, respectively. The ratio of C:T tended to be 1.98 and 1.50 times greater for the UASMS2 SNP compared to the E2FB SNP for steers and heifers, respectively (Table 1). This finding is in agreement with previously reported values [28] in which the C allele was much more common than the T allele for the UASMS2 SNP. The T allele is usually associated with fatter carcasses while the C allele yields leaner carcasses [29].

Table 2 reflects the Pearson correlation coefficients between MBV variables and model input and output variables. The MBV_REA_ was the variable with more significant correlations; it was highly correlated with FT, REA, and predicted EBF, DMI, and AFSBW_CT_. As expected, the MBV_CABMRB_ was correlated with FT, marbling score, and predicted EBF. The MBV_BTRFI_ had more significant correlations with input and output variables than the MBV_AllRFI_. Significant correlations between marbling MBV and marbling scores or intramuscular fat have been reported [20], suggesting that MBV scores can reliably be used to identify differences in carcass traits they were originally intended for. Thus, the correlations between MBV_REA_ and model input and output variables are expected to hold across different scenarios of production.

**Table 2:** Correlation matrix between molecular breeding value (MBV) scores and selected model input and output variables^1^.

Items	HH	fBW	HCW	FT	MRB	REA	EBF_C_	DOF	DMI_C_	DMI_HH_	DMR_C_	DMR_HH_	AFSBW_HH_	AFSBW_C_
**N**	1517	1517	1517	1517	1517	1517	1499	1517	1499	1499	1499	1499	1517	1499
**MBV**														
**CAB MRB**	-0.060	0.109	0.011	0.261	0.213	-0.177	0.326	0.016	-0.106	0.066	0.069	0.038	0.029	-0.203
	0.019	<0.0001	0.6762	<0.0001	<0.0001	<0.0001	<0.0001	0.5382	<0.0001	0.0104	0.0071	0.1446	0.2659	<0.0001
**ADG**	-0.031	0.069	0.032	0.060	0.046	-0.069	0.095	0.056	-0.009	0.014	0.011	0.006	-0.005	-0.035
	0.225	0.0072	0.2114	0.0187	0.0744	0.0072	0.0002	0.029	0.7243	0.5885	0.6642	0.8192	0.8341	0.1722
**HCW**	0.072	0.162	0.158	-0.068	0.063	0.144	-0.024	0.152	0.171	0.063	-0.043	-0.018	0.001	0.151
	0.0053	<0.0001	<0.0001	0.0077	0.0141	<0.0001	0.3496	<0.0001	<0.0001	0.0144	0.0994	0.4941	0.9706	<0.0001
**REA**	0.173	0.065	0.138	-0.335	-0.135	0.370	-0.365	0.199	0.258	0.029	-0.132	-0.085	0.047	0.353
	<0.0001	0.012	<0.0001	<0.0001	<0.0001	<0.0001	<0.0001	<0.0001	<0.0001	0.2637	<0.0001	0.0009	0.0688	<0.0001
**50 SNP MRB**	-0.062	0.112	0.020	0.217	0.189	-0.148	0.270	0.009	-0.084	0.066	0.088	0.061	0.041	-0.159
	0.0163	<0.0001	0.444	<0.0001	<0.0001	<0.0001	<0.0001	0.7312	0.0011	0.0112	0.0007	0.0183	0.1142	<0.0001
**50 SNP REA**	0.097	0.004	0.038	-0.152	-0.129	0.130	-0.180	0.061	0.090	-0.010	-0.036	-0.018	0.022	0.151
	0.0002	0.8848	0.1341	<0.0001	<0.0001	<0.0001	<0.0001	0.0173	0.0005	0.7118	0.1631	0.4923	0.392	<0.0001
**All MRB**	0.013	0.163	0.079	0.145	0.129	-0.041	0.189	0.135	0.006	0.097	0.010	-0.005	0.061	-0.060
	0.6228	<0.0001	0.0029	<0.0001	<0.0001	0.1182	<0.0001	<0.0001	0.8246	0.0003	0.6937	0.8466	0.02	0.023
**All REA**	0.066	0.100	0.101	-0.120	-0.071	0.159	-0.129	0.127	0.152	0.053	-0.046	-0.023	0.034	0.168
	0.0124	0.0001	0.0001	<0.0001	0.0069	<0.0001	<0.0001	<0.0001	<0.0001	0.0471	0.0828	0.3841	0.195	<0.0001
**All ADG**	-0.102	0.007	-0.044	0.199	0.121	-0.144	0.219	0.024	-0.132	0.012	0.009	-0.021	0.009	-0.180
	0.0001	0.7788	0.0927	<0.0001	<0.0001	<0.0001	<0.0001	0.3554	<0.0001	0.6499	0.7305	0.4242	0.7298	<0.0001
**All RFI**	0.091	0.067	0.099	-0.056	-0.047	0.106	-0.071	0.025	0.122	0.071	0.007	0.016	0.062	0.126
	0.0005	0.0111	0.0002	0.0337	0.0738	<0.0001	0.0076	0.347	<0.0001	0.007	0.8018	0.5373	0.0181	<0.0001
***Bos taurus* RFI**	-0.036	0.083	0.016	0.215	0.153	-0.094	0.245	0.111	-0.098	0.029	-0.009	-0.033	0.020	-0.149
	0.1674	0.0016	0.5398	<0.0001	<0.0001	0.0004	<0.0001	<0.0001	0.0002	0.2808	0.7219	0.2171	0.4384	<0.0001

^1^ For a given MBV, the first row indicates the Pearson correlation coefficient and the second row indicates the probability of H_0_: r = 0. HH = hip height; fBW = final (slaughter) BW, FT = fat thickness, MRB = marbling score, REA = ribeye area, EBF_C_ = predicted empty body fat using carcass traits, DOF = days on feed, DMI_C_ = predicted DMI using carcass traits, DMI_HH_ = predicted DMI using hip height information, AFSBW = predicted adjusted final shrunk BW at 28% empty body fat using carcass traits (AFSBW_CT_) or hip height (AFSBW_HH_), RFI = residual feed intake.

### Predicting days on feed

There was a moderate Pearson correlation (r = 0.73) between eDOF and DOF_CT_, but a low Pearson correlation (r = 0.44) between eDOF and DOF_HH_. There was an interaction (*P* < 0.001) between sex and DOF_HH_ or DOF_CT_. Although the impact of MBV variables was not uniform between heifers and steers for ΔDOF_HH_ and ΔDOF_CT_, MBV_REA_ and MBV marbling scores were able to explain at least an additional 8% of the variation of ΔDOF_HH_ and ΔDOF_CT_. Alone, the DOF_HH_ and DOF_CT_ explained 13.6 and 50.4% of the eDOF variation, respectively; and MBV_REA_ explained an additional 15.6 and 8.6% of the eDOF variation, respectively. Because DOF can be affected by ADG and AFSBW, as shown in Eq (8), it is possible that the inclusion of some MBV in the CVDS model as adjustment factors may increase its accuracy in predicting DOF. The initial SBW is a measured value; therefore, its measurement error is of little interest for this analysis. The main limitation of evaluating the relationship between ADG and MBV is that ADG depends on the predictions of DMI and the model inaccuracies might be related to its prediction of DMI rather than ADG per se, or a combination of both.

The stepwise regression of ΔADG_HH_ or ΔADG_CT_ on MBV and leptin genotypes yielded inconsistent relationships with little explanatory capacity, suggesting that either the CVDS model was not able to accurately predict ADG or there is little effect of the evaluated MBV and leptin genotype on ADG. The lack of correlation between ADG and MBV is consistent with previously reported values [20]. It seems that the MBV and leptin genotypes may influence the composition of the gain [47], not the ADG per se. In that sense, the partial efficiency of use of ME to NEg (i.e., k_g_) determined from gain composition might be of greater interest in the future because it can be influenced by the bioenergetics status of the animal [8, 48]. In fact, different leptin genotypes significantly altered the parameters of a power function for FT growth [19]. Therefore, given our current analysis, the effect of the evaluated MBV and leptin genotypes on DOF could only be attributed to AFSBW.

### Predicting AFSBW

The Pearson correlation between AFSBW_CT_ and AFSBW_HH_ was 0.48 (n = 670; *P* < 0.001) for heifers and 0.34 (n = 829; *P* < 0.001) for steers. For heifers and steers together, AFSBW_HH_ explained about 28% of the AFSBW_CT_ variation. A STEPWISE regression between ΔAFSBW and MBV scores indicated that MBV_REA_ could explain an additional 13.2% (*P* < 0.0001) of the variation in the AFSBW_CT_. Eq (9) (n = 1499, root of mean square error (**RMSE**) = 51.3 kg, r^2^ = 0.39) shows an interaction between sex and AFSBW_HH_, suggesting Eqs (1)–(3) may not be adequately accounting for the effects of sex on frame size and equivalent BW. It also suggested that MBV_REA_ was important in predicting AFSBW_CT_ and regardless of the sex, it would increase the average AFSBW_HH_ prediction by 44.5 kg. Assuming the ADG of 1.57 and 1.36 kg/d for steers and heifers, respectively observed in this study (Table 1), animals with greater MBV_REA_ (leaner) would need approximately an additional 30 d to reach the USDA low Choice grade. There was a tendency for an interaction between sex and MBV_50SNPMRB_ (*P* = 0.0922). The cross-validation analysis of 1,000 simulations confirmed a low precision of Eq (9), but high accuracy (Cb) (Table 3). The simulation also indicated the 95% quartile interval for the coefficient of MBV_REA_ to be within 38.6 to 50.2 kg.

$$AFSBW_{CT}=(312.8-175.9\times a)+(0.5354+0.392\times a)\times AFSBW_{HH}+44.5\times MBV_{REA}$$ (9)

**Table 3:** Adequacy statistics from the cross-validation simulations (n = 1,000) for selected equations.

Items^1^	Quartiles	Equations, units		
		(9), kg	(10), kg	(11), kg/d
Mean bias	---	-0.041	0.053	0
R^2^	2.5%	0.38	0.41	0.26
	97.5%	0.39	0.44	0.28
CCC	2.5%	0.554	0.596	0.42
	97.5%	0.564	0.613	0.43
Accuracy (Cb)	2.5%	0.894	0.919	0.82
	97.5%	0.905	0.933	0.83
Square root of MSEP	---	51.5	49.82	1.11
MSEP decomposition				
Mean bias, %	2.5%	0.001	0.001	0.001
	97.5%	1.43	1.26	1.36
Systematic bias, %	2.5%	0.002	0.021	0.002
	97.5%	1.458	1.41	1.53
Random variation, %	2.5%	97.9	98.0	97.8
	97.5%	99.9	99.9	99.9

^1^ MSEP = mean square error of prediction and CCC = concordance correlation coefficient.

Where AFSBW is adjusted final shrunk BW at 28% empty body fat predicted using carcass traits (AFSBW_CT_) or hip height (AFSBW_HH_), kg; MBV_REA_ is molecular breeding value for ribeye area; and a is an indicator variable for sex (0 = steers or 1 = heifers).

As expected [47], there was an effect (*P* < 0.001) of leptin genotypes on ΔAFSBW, but it became insignificant when combined with MBV_REA_. In fact, leptin genotypes (*P* < 0.001) and sex (*P* = 0.011) affected MBV_REA_ in which steers had a lower MBV_REA_ value than heifers (-0.494 and -0.434, respectively; *P* < 0.001) and the leptin genotype CCCC had the greatest MBV_REA_ value (-0.1331) compared to the other leptin genotypes. This was expected because both leptin SNP are part of the panel that makes up the MBV_REA_; they are mutually exclusive in the sense that when one is used the other one should not be included to avoid collinearity. Hence, because of the association between MBV_REA_ and leptin genotypes only MBV_REA_ was retained in Eq (9).

Eq (10) (n = 1499, RMSE = 49 kg, r^2^ = 0.45) uses HH and initial BW associated with MBV_REA_ and the MBV_CABMRB_. There was a slight decrease in the error of the prediction and an increase in the precision compared to Eq (9). Some variation, which can be accounted for in the marbling panel, is not accounted for by the REA panel, so the inclusion of these MBV simultaneously suggested that both might be significant, although the sign of the effect is opposite. The range of MBV_CABMRB_ in this dataset was essentially 100 points, so the difference between the best and the worst would vary AFSBW by 25 kg.

$$\begin{array}{l} AFSBW_{CT}=(-1184.7+15758\times a)+(34.292-270.98\times a)\times HH-\\ (2.155+53.347\times a)\times iSBW+(0.00888+0.9126\times a)\times HH\times iSBW-\\ (0.165-1.152\times a)\times HH^{2}+(0.00009-0.00387\times a)\times HH^{2}\times iSBW-\\ 0.257\times MBV_{CABMRB}+39.04\times MBV_{REA} \end{array}$$ (10)

Where AFSBW_CT_ is the adjusted final shrunk body weight at 28% empty body fat using carcass traits, kg; HH is hip height, cm; iSBW is initial shrunk body weight, kg; MBV_CABMRB_ and MBV_REA_ are the molecular breeding value for marbling for Angus cattle and REA, respectively; and a is an indicator variable for sex (0 = steers or 1 = heifers).

The cross-validation of Eq (10) (Table 3) suggested a slight improvement in the adequacy statistics, but most of the errors, as expected, were random errors due to the large variation and incomplete correlation of the variables. The simulations also indicated that coefficient for MBV_CABMRB_ and MBV_REA_ were likely to vary (95% quartile interval) from -0.457 to -0.057 and 31.9 to 46.2, respectively.

### Predicting dry matter required

There was no significant correlation between DMR and MBV for RFI (*P* > 0.21). This was expected based on previous analysis [49] of which no phenotypic correlation between RFI and DMR during the growing and finishing phases of beef cattle was observed. Because mean BW and ADG usually does not explain all the variation in DMI, we would expect a phenotypic correlation between observed DMI and RFI (0.42 < r < 0.74; [15, 50]). Some [51] have suggested that nutritional models cannot satisfactorily determine individual DMI and RFI of group-fed cattle. Others [16] have indicated strong correlations (r > 0.89) between RFI and predicted intake difference (**PID** = observed DMI—DMR), suggesting that PID would be more capable to identify animals with low DMI and slow ADG as inefficient compared to RFI. This lack of correlation between DMR and MBV RFI suggest that no additional variation of DMR can be explained by the MBV RFI, although one would expect some correlation between PID and RFI (0.58 < r < 0.78; [49]). For steers, DMR explained 24% of DMI variation. Nonetheless, MBV_REA_ was able to explain an additional 6.5% of the ΔDMI variation as shown by the correlation between MBV_REA_ and DMR (Table 2). As shown in Eq (11) (n = 1499, RMSE = 1.11, r^2^ = 0.28), as MBV_REA_ increased one unit, DMR tended to decrease by 0.6 kg/d. The result of the cross-validation simulation of Eq (11) is shown in Table 3. Despite the low precision, a high accuracy can be obtained and most of the variation in the MSEP is due to incomplete co-variation. Based on the cross-validation, the impact of the MBV_REA_ is likely to range from -0.74 to -0.49 kg/d.

$$DMR=(-3.41+6.93\times a)+(1.48-1.054\times a)\times pDMI-0.606\times MBV_{REA}$$ (11)

Where DMR is dry matter required, kg/d; pDMI is predicted DMI, kg/d; and MBV_REA_ is the molecular breeding value for REA, and a is an indicator variable for sex (0 = steers or 1 = heifers).

Some relationship between DMR and MBV is expected due to moderate heritability reported for DMR computed with either carcass traits (0.35) or carcass ultrasound (0.32) information [52], and the high genetic correlation between DMR and DMI (greater than 0.98 [52] and greater than 0.79 [53]).

Because individual DMI is rarely available under feedlot conditions, DMR using AFSBW_CT_ was assumed as its best predictor. An alternative equation to estimate DMI [38] was used instead of the NRC’s empirical equation [8]. The correlation between estimated DMI using two empirical equations ([8] and [38]) was moderate (r = 0.582), but MBV_REA_ was still significant in explaining variation in DMR, as shown in Eq (12) (n = 1499, RMSE = 1.17, r^2^ = 0.20). As MBV_REA_ increased one unit, DMR tended to decrease by 0.45 kg/d. This results is similar to that obtained with Eq (11).

$$DMR=(0.71+3.01\times a)+(0.959-0.453\times a)\times pDMI_{\text{End BW}}-0.45\times MBV_{REA}$$ (12)

Where DMR is dry matter required, kg/d; pDMI_End BW_ is predicted DMI, kg/d; and MBV_REA_ is the molecular breeding value for REA, and a is an indicator variable for sex (0 = steers or 1 = heifers).

An analysis [54] indicated that MBV evaluated in breeds that were not included in the training set had genetic correlations around zero, suggesting that MBV may have limited prediction accuracy for different breeds. The diversity of animals fed in feedlots may have improved the effectiveness of MBV scores obtained in this study, suggesting that MBV obtained for specific breeds may further improve the predictability of animal performance by nutrition models when distinct genetic groups are taken into account. The centerpiece of the CVDS growth model is that USDA low Choice is achieved when animals reach 28.6% EBF [30]. Though this EBF endpoint may change [33], depending on sex and breed type, scarce information is available in the literature. The present analysis can be tailored to accommodate different EBF endpoints.

In conclusion, our analysis indicates that it is possible (and desirable) to incorporate genomic information into mathematical nutrition models to enhance their predictability by accounting for individual makeup (i.e., genetic) differences. This incorporation, however, may require profound modifications of nutrition models to accommodate new concepts, such as genome wide associated studies [21, 55], to remove genetic/genomic effects that are embedded in current growth model coefficients, and to separate intrinsic interrelationship among currently accounted for variables. Building upon the advancements in genomics and nutrition modeling, adding genomic information to nutrition models (e.g., growth models) is critical to enhance the characterization of the animal and its biological fingerprint in order to produce high quality meat in a more sustainable way.

## Supporting Information

S1 File Inputs and computer simulated variables are provided in the S1 File. (ZIP)

## Abbreviations

ADG: Average daily gain, kg/d
AFSBW: Adjusted final shrunk body weight, kg
BCS: Body condition score
BW: Body weight, kg
Cb: Accuracy
CCC: Concordance correlation coefficient
CVDS: Cattle Value Discovery System
DMI: Dry matter intake, kg/d
DMR: Dry matter required, kd/d
DOF: Days on feed
EBF: Empty body fat, %
EBV: Estimated breeding value
EBW: Empty body weight, kg
FT: Fat thickness, cm
HCW: Hot carcass weight, kg
HH: Hip height, cm
MBV: Molecular breeding value
ME: Metabolizable energy of the diet, Mcal/kg
MSEP: Mean square error of prediction
NEg: Net energy of the diet available for growth, Mcal/kg
NRC: National Research Council
PID: Predicted intake difference, kg/d
QG: Quality grade
REA: Ribeye area, cm^2^
RFI: Residual feed intake, kg/d
RMSE: Root of mean square error
RMSEP: Root of mean square error of prediction
SBW: Shrunk body weight, kg
SNP: Single nucleotide polymorphism
USDA: United States Department of Agriculture
YG: Yield grade
